# Immediate Loading on Tapered Versus Straight Multiple Implants: A 3‐Year Follow‐Up of a Randomized Clinical Trial

**DOI:** 10.1111/cid.70147

**Published:** 2026-04-14

**Authors:** Young Woo Song, Seung‐Hyun Park, Franz J. Strauss, Sung Jin Yoo, Jin‐Young Park, Jae‐Kook Cha, Ui‐Won Jung

**Affiliations:** ^1^ Department of Periodontology Dental Hospital, Veterans Health Service Medical Center Seoul South Korea; ^2^ Department of Periodontology Research Institute for Periodontal Regeneration, Yonsei University College of Dentistry Seoul South Korea; ^3^ Clinic of Reconstructive Dentistry, Center of Dental Medicine, University of Zurich Zurich Switzerland; ^4^ Health Science Faculty, Universidad Autonoma de Chile Santiago Chile

**Keywords:** dental implant, immediate loading protocol, implant design, implant survival, marginal bone level, partial edentulism

## Abstract

**Objective:**

To evaluate the clinical and radiographic outcomes at 3 years following immediate loading of tapered implants (TI) versus straight implants (SI) from a randomized controlled trial (RCT).

**Materials and Methods:**

Of the 41 patients who received 93 implants in the original RCT (TI group: 23 patients, 50 implants; SI group: 18 patients, 43 implants), those who completed 3‐year follow‐up were included in this analysis. Implant survival and success rates, as well as changes in marginal bone loss (MBL), were assessed and compared using Kaplan–Meier curves, Cox proportional hazards models, and linear mixed‐effects models.

**Results:**

A total of 28 patients with 61 implants were evaluated (TI group: 15 patients, 32 implants; SI group: 13 patients, 29 implants). At 3 years post‐immediate loading, cumulative survival rates at the patient level were 95.8% for the TI group and 78.3% for the SI group (intergroup *p* = 0.109). At the implant level, survival rates were 96.2% for TI and 90.0% for SI (intergroup *p* = 0.351). All implant failures occurred within the first year (1 implant in TI; 5 implants in SI), and no further losses were recorded thereafter. However, two TI implants in a single patient exhibited progressive peri‐implantitis during follow‐up. Mean MBL values were 0.61 ± 1.70 mm for the TI group and 0.53 ± 0.53 mm for the SI group, with no statistically significant differences between groups (intergroup *p* = 0.428).

**Conclusion:**

It could be cautiously concluded that the TI rather than the SI might be a more predictable option in terms of achieving an optimized initial stability for immediate loading in the posterior region. Once osseointegration was successfully achieved, however, the macro‐design of the implant might not have an influence on the clinical and radiographic outcomes including the osseointegrated state during the 3 years of observational period.

**Trail Registration:** KCT0002489; https://cris.nih.go.kr/cris/search/detailSearch.do?search_lang=E&focus=reset_12&search_page=L&pageSize=10&page=undefined&seq=19032&status=5&seq_group=8332

## 1 Introduction

Immediate loading of dental implants is recognized for reducing treatment time and improving patient satisfaction [1, 2, 3, 4]. Traditionally, clinicians have delayed prosthetic loading for several months to ensure successful osseointegration prior to prosthesis delivery, particularly for machined‐surface implants [5, 6]. However, advancements in implant surface treatments that increase hydrophilicity have shortened the osseointegration period [7, 8], enabling earlier functional loading [1, 9, 10]. Alongside enhanced surface properties, advances in implant fixture topography have allowed clinicians to achieve higher primary stability more readily, making immediate loading—placement of the prosthesis within a week after implant placement—a viable option in many clinical situations [5, 6, 11, 12, 13, 14] even when delivering the final restoration [15, 16].

Given that the feasibility of immediate loading is highly dependent on the primary stability of the implant, the design of the implant fixture is considered a critical factor influencing immediate loading success [11]. Among various designs, tapered implants with self‐tapping grooves are known to offer greater primary stability compared to straight implants without such grooves. Nevertheless, to date, no randomized controlled trial (RCT) has directly compared these two designs under immediate loading conditions in the posterior dentition.

Previously, a 1‐year outcome from a RCT investigating immediate loading in partially edentulous posterior regions with both tapered (TI) and straight (SI) implants was published [11]. That study reported high survival and success rates for tapered implants (96%) and more modest survival rates for straight implants (86%) while both implant types showed minimal marginal bone loss (MBL). Notably, TIs featuring an apical self‐tapping configuration achieved significantly higher insertion torque at placement compared to the SIs, suggesting superior initial mechanical stability.

Given the importance of long‐term data in implant dentistry, extended follow‐up of this cohort is essential to determine the durability of outcomes and to clarify the role of implant design on immediate loading in the posterior region, an area where data remain limited. Therefore, the present study aimed to investigate the 3‐year follow‐up outcomes from the previous RCT comparing immediate loading tapered and straight implants.

## 2 Materials and Methods

### 2.1 Study Design and Population

The RCT was conducted from September 2017 to March 2020 by periodontists and prosthodontists affiliated with the Yonsei University College of Dentistry, under approval from the Institutional Review Board of Yonsei University Dental Hospital (No. 2‐2016‐0047) and registered with the Clinical Research Information Service of the National Research Institute of Health in South Korea (https://cris.nih.go.kr; Registration No, KCT0002489). The details of the research design and sample size power calculation are described in a previously published article [11], and the current study adhered to the CONSORT guidelines.

Based on the previously reported inclusion and exclusion criteria, a total of 48 patients requiring placement of two or more implants in the posterior maxilla or mandible were recruited and randomly assigned to either the TI group (*n* = 24) or the SI group (*n* = 24). In the TI group, tapered implants with a self‐tapping cutting edge (Yuhan Evertis SI, Shinhung, Seoul, South Korea) were used, while in the SI group, straight implants without a cutting edge (Straumann Bone Level, Straumann, Basel, Switzerland) were placed. Both the TI and SI fixtures had a sandblasted, large‐grit, and acid‐etched (SLA) surface. Of the 41 subjects who received 93 implants (TI group: 23 patients, 50 implants; SI group: 18 patients, 43 implants) and completed the original trial [11], the present follow‐up study included those patients who were followed up for 3 years after immediate loading.

### 2.2 Clinical Protocol and Data Collection

According to the previously described research protocol [11], patients received multiple implant placements, with guided bone regeneration performed as needed. A provisional splinted prosthesis made of polymethylmethacrylate was delivered within 7 days after surgery. After 1 year, a definitive prosthesis identical to the provisional prosthesis was placed, and patients subsequently attended periodic recall checkups at 3‐to‐6‐month intervals. All clinical and radiographic data were collected from the electronic dental record system for this study.

### 2.3 Outcome Variables

#### 2.3.1 Implant Survival and Failure Analysis

The survival rate was defined as the percentage of implants that remained intraorally at the time of examination without any indication for fixture removal. Conversely, failure was defined as the absence of the implant from the oral cavity for any reason.

#### 2.3.2 Composite Treatment Success of Immediate Loading Protocol

The success of the immediate loading protocol was determined at the implant level based on the following criteria:

– Installation of the fixture with an insertion torque of 30 Ncm or higher [5, 17]
– Maintenance of osseointegration over time following immediate loading [5]
– Annual MBL of less than 0.2 mm after immediate loading [5, 17, 18]

Implants that met all of these criteria were considered successful regarding immediate loading; otherwise, the treatment protocol was considered unsuccessful.

#### 2.3.3 Peri‐Implant Marginal Bone Level Analysis

To evaluate changes in marginal bone level over time, serial periapical radiographs were collected at the most recent follow‐up and at 1 year after implant loading. These radiographs were taken using a standardized extension cone paralleling technique with a digital sensor positioning device (Rinn; Dentsply Sirona, Charlotte, NC, USA). Peri‐implant MBL, defined as the vertical distance (in mm) between the implant shoulder and the first bone‐to‐implant contact, was measured at each time point using dedicated software (ZeTTA PACS Viewer version 2.0.0.4 BN2, TaeYoung Soft, Seoul, South Korea), and the difference between measurements was recorded as the change in MBL over time. All measurements were calibrated to the actual implant fixture length and were performed by a single experienced examiner (S.J.Y.) on both the mesial and distal sides of the implants [19], and the average of these two values was used as the representative MBL for each implant. Annual MBL (mm/year) was then calculated by dividing the measured MBL by the observation period.

### 2.4 Statistical Analysis

Statistical analyses were performed using SPSS (version 26; IBM, Armonk, NY, USA) and R (version 4.4.0; R Foundation for Statistical Computing, Vienna, Austria). Cumulative survival rates at both the implant and patient levels were evaluated using the Kaplan–Meier method, and differences between the TI and SI groups were assessed using Cox proportional hazards regression.

To account for the clustering of multiple implants within the same patient, annualized marginal bone loss was analyzed using a linear mixed‐effects model (LMM) with a patient‐level random intercept. The fixed‐effects structure included implant group (TI or SI), observation time, and their interaction to assess potential time‐dependent differences between groups. Estimated marginal means with corresponding 95% confidence intervals were computed using the *emmeans* package in R.

All R code used for the analyses is provided in the Supporting Information. Statistical significance was set at *p* < 0.05.

## 3 Results

### 3.1 Demographic Information of the Patients and Implants

**FIGURE 1:**
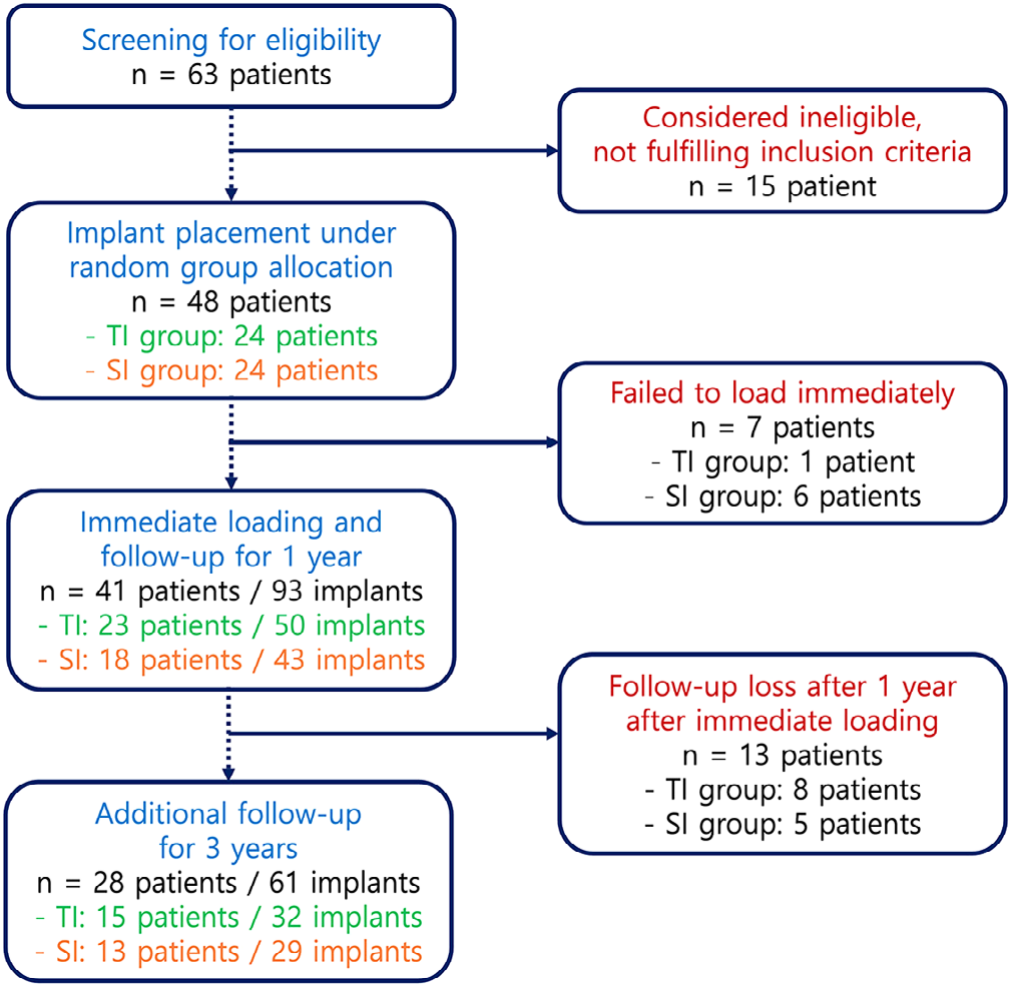
CONSORT Flowchart.

Among the 41 patients who had completed the clinical trial, 13 (8 TI‐group and 5 SI‐group) patients could not be followed‐up due to their refusal of re‐call visit after finishing the original research protocol, stating that they had no discomfort. As a result, a total of 61 implants placed in 28 patients were ultimately included in the analysis (TI group: 32 implants in 15 subjects; SI group: 29 implants in 13 subjects) (Figure 1). A sensitivity analysis was carried out by statistically comparing the demographic data between the followed‐up and drop‐out subjects, and it was confirmed that there was no statistically significant difference (*p* > 0.05), implying that the attrition bias was not present in the present study.

**FIGURE 2:**
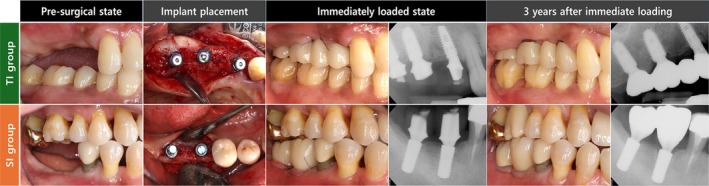
Representative clinical photographs and radiographs taken for 3 years of follow‐up observation.

The mean ages were 64.80 ± 9.74 years for the TI group and 63.00 ± 9.21 years for the SI group. In both groups, most patients received two implants, while two patients in each group received three or more implants. Demographic information is summarized in Table 1, and representative clinical photographs and radiographs are shown in Figure 2.

**TABLE 1:** Demographic information.

Demographics of patients included in the present study	TI group	SI group
Number of patients	15	13
Age in years (mean ± standard deviation)	61.80 ± 9.74	63.00 ± 9.21
Gender		
Male	8	9
Female	7	4

Demographics of implants included in the present study	TI group	SI group
Number of implants	32	29
Length		
7 mm	1	—
8–8.5 mm	11	3
10 mm	19	25
11.5–12 mm	1	1
Diameter		
3.3–4.1 mm	5	10
4.5 mm	5	0
4.8–5 mm	22	19

Demographics of treated site (number of implants)	TI group	SI group
Jaw type		
Maxilla	10	4
Mandible	22	25
Whether guided bone regeneration accompanied implant placement		
Yes	4	5
No	28	24

Demographics of provisional prostheses	TI group	SI group
Design of supraconstruction (number of patients)		
2‐unit abutment	9	7
3‐unit abutment	4	5
4‐unit abutment	2	0
5‐unit abutment	0	1
Material of final prosthesis (number of patients)		
Zirconia	8	10
Gold	2	1
Porcelain‐fused metal/porcelain‐fused gold	5	2
Retention type of final prosthesis (number of patients)		
Screw‐cement‐retained	14	13
Cement‐retained	1	0

### 3.2 Implant Survival and Failure Analysis

**FIGURE 3:**
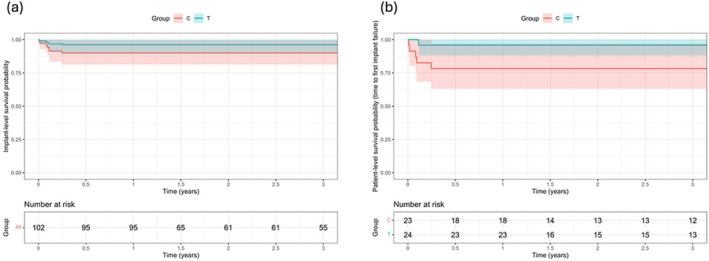
Kaplan–Meier survival curves for dental implants during the 3‐year observation period. No statistically significant difference was observed between the two groups (*p* > 0.05). (a) Implant‐level survival probability. The cumulative survival rate of the test group (T, blue line) and the control group (C, red line) is presented with shaded areas representing the 95% confidence intervals. (b) Patient‐level survival probability (time to first implant failure). The analysis accounts for the first occurrence of implant failure per patient. The “Number at risk” tables below each graph indicate the remaining number of implants (a) and patients (b) at each time interval.

As described in the previous publication, a total of 2 implants in the TI group and 5 implants in the SI group were removed within the first 3 months after immediate loading due to postsurgical infection or early failure of osseointegration [11]. No additional implant failures occurred in either group during the subsequent 3‐year observation period. As a result, the cumulative implant survival rates at 3 years after loading were 96.2% at the implant level and 95.8% at the patient level for the TI group, compared to 90.0% (implant level) and 78.3% (patient level) for the SI group (Table 2). The difference between the two groups, assessed using the Cox proportional hazard model analysis, showed a non‐significant trend toward a difference between the groups (*p* = 0.109) (Figure 3).

**TABLE 2:** Implant survival analysis: Kaplan–Meier survival rates at implant‐ and patient‐level.

	TI group	SI group	*p*
Implant‐level			
At 3 years (%)	96.2 (90.1, 99.5)	90.0 (81.4, 99.5)	0.351
Patient‐level			
At 3 years (%)	95.8 (88.2, 100)	78.3 (63.1, 97.1)	0.109

*Note:* Mean values with 95% confidence intervals were presented. For the implant‐level analysis, a Cox proportional hazards model with robust standard errors clustered by patient was applied.

### 3.3 Composite Treatment Success of Immediate Loading Protocol

As previously reported [11], 2 SI‐group implants could not be immediately loaded because their insertion torque values were lower than 30 Ncm, and 7 implants (2 in the TI‐group and 5 in the SI‐group) experienced early failure within the first year after immediate loading. In addition, 2 TI‐group implants and 6 SI‐group implants exhibited an annual MBL exceeding 0.2 mm. Consequently, over the 3‐year follow‐up period, a total of 13 SI‐group implants and 4 TI‐group implants were classified as unsuccessful with respect to the immediate loading protocol. The outcomes are summarized in Table 3.

**TABLE 3:** Implants that failed to successfully perform the immediate loading protocol.

	TI group	SI group
Total number of unsuccessful implants	4	13
Subtotal number of unsuccessful implants		
Failed to achieve an insertion torque of 30 Ncm or higher	0	2^a^
Failed to maintain osseointegrated state over time after immediate loading^b^	Early: 2 Late: 0	Early: 5 Late: 0
Annual MBL of 0.2 mm or more after immediate loading	2	6

^a^ Consequently, conventional loading protocols were applied.

^b^ Consequently, the implants were removed: Early, within 1 year; Late, after 1 yearMBL: marginal bone loss.

### 3.4 Peri‐Implant Marginal Bone Level Analysis

At 1 year after immediate loading, the TI group showed slightly lower MBL values (0.13 ± 0.36 mm) compared to the SI group (0.25 ± 0.53 mm). When assessing marginal bone levels based on the most recent periapical radiograph, the TI group exhibited a mean bone loss of 0.61 ± 1.70 mm and 0.53 ± 0.53 mm in the SI group, with no significant differences (Table 4a).

**TABLE 4a:** Peri‐implant marginal bone loss analysis: Mean marginal bone loss occurred during each period.

	TI group	SI group	*p*
MBL over the observational period of 3 years	0.48 ± 1.47 mm	0.28 ± 0.34 mm	0.276
MBL measured at 1 year after immediate loading	0.13 ± 0.36 mm	0.25 ± 0.53 mm	0.542
MBL measured from the latest periapical x‐ray	0.61 ± 1.70 mm	0.53 ± 0.53 mm	0.428

*Note:* For inter‐group comparison, a linear mixed model analysis with the observational period as a fixed factor and patient as a random factor was used.

Abbreviation: MBL: marginal bone loss.

At the 3‐year follow‐up, the estimated mean MBL values were 0.23 ± 0.42 mm for the TI group and 0.50 ± 0.64 mm for the SI group (*p* = 0.729). The annual MBL was 0.13 ± 0.13 mm/year for the TI group and 0.11 ± 0.14 mm/year for the SI group, with no statistically significant difference between the groups (*p* = 0.868) (Table 4b).

**TABLE 4b:** Peri‐implant marginal bone loss analysis: Estimated mean values of marginal bone loss at 1 and 3 years and annual bone loss.

	TI group	SI group	*p*
Estimated means at 1 year	0.13 ± 0.18 mm	0.21 ± 0.20 mm	0.543
Estimated means at 3 years	0.23 ± 0.42 mm	0.50 ± 0.64 mm	0.729
Annual marginal bone loss	0.13 ± 0.13 mm/year	0.11 ± 0.14 mm/year	0.868

*Note:* Group‐specific estimated means were calculated using a linear mixed‐effects model that incorporated the observational period as a fixed factor and the patient as a random factor.

### 3.5 Biologic and Mechanical Complications

**FIGURE 4:**
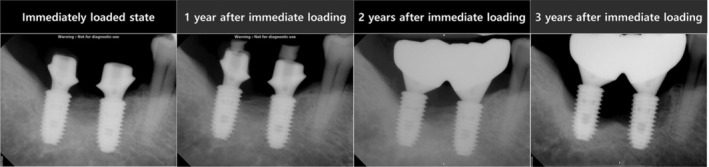
Serially taken periapical radiographs of the ailing implants in the TI group.

A biologic complication, manifesting as peri‐implant disease, occurred in only 1 TI‐group patient who had 2 implants supporting a splinted prosthesis at the mandibular first and second molar sites. These implants had already demonstrated 0.48 mm (first molar) and 1.79 mm (second molar) of marginal bone losses 1 year after immediate loading, of which the bone losses reached 7 mm on both sites at 3 years follow‐up (Figure 4).

Regarding prosthetic complications, 2 patients in the SI group experienced issues: one had exfoliation of a 2‐unit bridge, and the other had a partial crown fracture. Both prostheses were immediately restored once the complications were clinically identified.

The complications are summarized in Table 5.

**TABLE 5:** Biologic and mechanical complications occurred in both groups.

	TI group	SI group
Biologic complication (number of implants) Peri‐implantitis Mechanical complication (number of implants)	2	0
Exfoliation of crown	0	2
Partial fracture of crown	0	2

## 4 Discussion

The present study evaluated the 3‐year follow‐up outcomes of a cohort originally enrolled in a two‐armed RCT investigating immediate loading of multiple implants placed in the partially edentulous posterior jaw. The main findings were: (i) a trend toward lower survival rates for straight implants compared to tapered implants particularly at the patient level (~96% vs. ~78%) and (ii) no clinically meaningful differences in MBL between the implant fixture macro‐designs.

Survival in the tapered implant group remained approximately 96% at both the patient and implant levels. In contrast, survival in the straight implant group decreased to ~90% at the implant level and ~78% at the patient level. A systematic review assessing mid‐term (1–5 years) outcomes of immediate loading after delayed or late implant placement reported a weighted survival rate of 97.9% [6]. Importantly, that systematic review also noted that survival rates for immediately loaded implants ranged more widely (83.3% to 100%), compared to conventional loading (95.5% to 100%) [6], which corresponds to the variability observed in the straight implant group in this study.

Similarly, a retrospective study documented substantially lower 5‐year survival for immediately loaded implants (60%) compared with conventionally loaded implants (99%) [20]. The variability observed here is in line with findings for immediately placed and immediately loaded single implants. A recent systematic review with meta‐analysis reported a 3‐year survival of 97.5%, but when restricted to RCTs, survival decreased to 93% (range, 85%–96%), closely mirroring the present results, especially for straight implants.

Differences in reported survival across studies likely reflect heterogeneity in study design and risk of bias. Non‐randomized studies may over‐ or underestimate treatment effectiveness due to selection bias, lack of blinding, or uncontrolled confounders. RCTs therefore remain the gold standard for evaluating treatment efficacy and informing evidence‐based decision‐making [21].

Regarding MBL, both implant groups exhibited less than 1 mm of bone loss over the 3‐year period, consistent with previous reports evaluating immediate loading after 3 to 5 years in the posterior region [22, 23]. Only three implants in each group exhibited MBL greater than 1 mm. Among these, two tapered implants showed progressive and substantial bone loss: initial 1‐year losses of 0.48 mm (first molar) and 1.79 mm (second molar) increased by 6.53 mm and 5.22 mm, respectively, reaching approximately 7 mm. Although such cases might be classified as failures warranting removal based on prior literature, these implants were maintained through supportive therapy and local antibiotic application. Although such cases might be classified as failures warranting removal based on prior literature [24, 25, 26, 27], these implants were maintained through supportive therapy and local antibiotic application [28, 29].

No fixtures in either group experienced any biological complications, except for the 2 TI‐group implants which were diagnosed peri‐implantitis according to the recently published consensus report [30]. Since the affected fixtures were present intraorally, these were classified as survival at the time point of 3 years post‐immediate loading. Nevertheless, the extent of bone resorption exceeding 50%, reaching 70% of the fixture length, and therefore according to the clinical recommendation suggested by previous publications [24, 25, 26, 27], fixture removal might have been considered. This implies that the decision of keeping these two implants intraorally instead of removing them has affected the clinical and radiographic data, increasing the survival rate as well as the marginal bone loss in the TI group.

Looking back at the forementioned diseased TI‐group case, not only was the patient non‐compliant with oral hygiene, but the two severely compromised TI implants also exhibited a convex emergence profile with an emergence angle exceeding 30°, particularly in the interproximal region. Preclinical evidence indicates that steeper emergence angles may impair self‐cleansability, promote plaque accumulation, increase MBL, and disrupt the integrity of the junctional epithelium of the implant supracrestal complex [31, 32]. Clinical studies similarly suggest that concave profiles [33, 34] or emergence angles below 30°–40° are associated with improved oral hygiene, reduced bleeding on probing, and lower MBL compared with convex profiles or emergence angles exceeding 30°–40° [35, 36, 37, 38, 39, 40, 41]. Therefore, it is probable that self‐cleansing was inadequate due to the limited interproximal space of the splinted crown, eventually promoting the development of peri‐implantitis.

Mechanical complications involving prosthetic components occurred in 6 out of 29 SI‐group fixtures (20.7%), which is similar to findings from previous 3‐year follow‐up studies [42], whereas none of the TI‐group implants experienced such issues. It can be cautiously speculated that these prosthetic complications in the SI group were attributable to the parafunctional habits of two SI‐group patients.

Notably, beyond the first year, none of the 61 implants demonstrated osseointegration failure while functioning. Previous studies have shown that most failures of immediately loaded implants occur in the early phase of healing [43, 44, 45, 46]. As previously reported for this cohort, five straight implants failed to osseointegrate under immediate loading during the first year, and two additional straight implants were not eligible for immediate loading due to insufficient insertion torque (< 30 Ncm). Two tapered implants were removed early due to postsurgical infection at the GBR site.

Collectively, these observations suggest that implant macro‐design may influence the success of immediate loading, especially regarding successfully achieving an optimized level of insertion torque of 30Ncm or higher. As previously reported, the screw‐shaped design of TI‐group implants contributed to higher primary stability compared to SI‐group implants [11], providing a more favorable environment for withstanding occlusal forces just days after implant placement [11, 12, 47]. The secondary stability, however, highly depends on the surface hydrophilicity rather than the macro‐design, and both TI‐ and SI‐group implants would be expected to achieve successful osseointegration within 3 months, as supported by previous preclinical and clinical studies [8, 47]. Although several publications have indicated that implant design may influence stress distribution to the surrounding bone and potentially relate to marginal bone resorption due to occlusal stress [48, 49, 50, 51]. such bone loss did not appear in the present study. As stated earlier, the only instance of severe marginal bone resorption leading to fixture removal was attributable to uncontrolled peri‐implantitis, primarily due to unfavorable abutment design.

The present research proposed the composite treatment success criteria of immediate loading protocol, which had not been suggested in the literature. Despite the lack of evidence, each individual criterion was determined based on previous articles [5, 17, 18], from the classic to the recent consensus, in order to enhance the persuasiveness. The 3‐year success of the immediate loading protocol differed markedly between groups: failures were more than twice as frequent in the straight implant group (13 implants) than in the tapered implant group (4 implants). Notably, more than half of the unsuccessful straight implants (7 of 13) failed due to early events related to insufficient primary stability. These findings suggest that tapered implants may provide more predictable outcomes than straight implants when immediate loading is planned.

The main limitation of the present research was the drop‐out rate at 3 years over 30%. Even though it was statistically confirmed that the attrition bias could be ignored, the statistical power to detect significant differences between the groups would have been higher if more patients were followed up. It is necessary to add the data of these drop‐out patients to future research of longer follow‐up period.

## 5 Conclusion

Within the limitations of this study, although both the tapered and straight designs showed comparable 3‐year outcomes, it could be cautiously concluded that the tapered implants may offer a more predictable initial stability for immediate loading in the posterior region. Once osseointegration was successfully achieved, however, whether the macro‐design of the implant was tapered or straight did not seem to have influenced the osseointegrated state of the implant during the 3 years of observational period.

## Author Contributions

Conceptualization: Ui‐Won Jung. Data curation: Young Woo Song. Formal analysis: Seung‐Hyun Park (equal) and Franz J. Strauss (equal). Funding acquisition: Ui‐Won Jung. Investigation: Young Woo Song (lead) and Sung Jin Yoo (supporting). Methodology: Ui‐Won Jung and Jae‐Kook Cha. Project administration: Ui‐Won Jung. Resources: Jin‐Young Park (equal) and Seung‐Hyun Park (equal). Software: Seung‐Hyun Park. Supervision: Ui‐Won Jung (lead) and Jae‐Kook Cha (supporing). Validation: Ui‐Won Jung (lead) and Jae‐Kook Cha (supporing). Visualization: Young Woo Song. Writing – original draft: Young Woo Song (equal) and Seung‐Hyun Park (equal). Writing – review and editing: Ui‐Won Jung (lead), Jin‐Young Park (supporting), Jae‐Kook Cha (supporting), and Franz J. Strauss (supporting).

## Funding

This research was supported by a grant of the Korea Health Technology R&D Project through the Korea Health Industry Development Institute (KHIDI), funded by the Ministry of Health & Welfare, Republic of Korea (Grant No. HI17C1997).

## Conflicts of Interest

The authors declare no conflicts of interest.

## Supporting information


**Data S1:** cid70147‐sup‐0001‐Supinfo.docx.

## Data Availability

The data that support the findings of this study are available from the corresponding author upon reasonable request.
