# Supplementary material for: Immediate Loading on Tapered Versus Straight Multiple Implants: A 3‐Year Follow‐Up of a Randomized Clinical Trial
Source: Clin Implant Dent Relat Res. 2026 Apr 14;28(2):e70147. doi: 10.1111/cid.70147 (PMC13080229; doi:10.1111/cid.70147)
Supplement: Supplementary file 1 — Data S1: cid70147‐sup‐0001‐Supinfo.docx. [file CID-28-0-s001.docx]

**[Supplementary methods: R codes used for statistical analyses]**

**[implant level survival rate: p-value based on cox proportional hazard model]**

fit_impl_robust <- coxph(Surv(time_adj, event) ~ group,

data = df_impl, ties = "efron",

cluster = patient)

sf_impl_robust <- survfit(fit_impl_robust,

newdata = data.frame(group = levels(df_impl$group)))

**[implant level survival rate: mean, 95% CI]**

ss3_impl <- summary(sf_impl_robust, times = 3.0, extend = TRUE)

km3_impl <- tibble(

group = if (!is.null(ss3_impl$strata)) sub("^group=", "", ss3_impl$strata)

else levels(df_impl$group)[1],

time = ss3_impl$time,

surv = ss3_impl$surv,

lower = ss3_impl$lower,

upper = ss3_impl$upper,

n_risk = ss3_impl$n.risk,

n_event = ss3_impl$n.event

)

**[patient level survival rate: p-value based on cox proportional hazard model]**

fit_pat <- coxph(Surv(time, event) ~ group,

data = df_pat, ties = "efron")

**[patient level survival rate: mean, 95% CI]**

sf_pat <- survfit(Surv(time, event) ~ group, data = df_pat)

for (tt in c(1.0, 3.0)) {

ss_t <- summary(sf_pat, times = tt, extend = TRUE)

km_t <- tibble(

group = if (!is.null(ss_t$strata)) sub("^group=", "", ss_t$strata) else levels(df_pat$group)[1],

time = ss_t$time, surv = ss_t$surv,

lower = ss_t$lower, upper = ss_t$upper,

n_risk = ss_t$n.risk, n_event = ss_t$n.event

)

cat("\n[Patient-level] ", tt, " years\n", sep = "")

print(km_t)

}

**[marginal bone loss: mean, SD, estimated means]**

m_lmm <- lmer(rblfinal ~ group * observation + (1 | patient), data = df)

em1_3 <- emmeans(m_lmm, ~ group | observation, at = list(observation = c(1,3)))
